# Trend in the Prevalence of Non-Daily Smoking and Their Relationship with Mental Health Using the Korea Health and Nutrition Examination Survey

**DOI:** 10.3390/ijerph17103396

**Published:** 2020-05-13

**Authors:** Yunna Kwan, Hye Sim Kim, Dae Ryong Kang, Tae Hui Kim

**Affiliations:** 1 Department of psychiatry, Wonju Severance Christian Hospital, Wonju 26426, Korea; kwbl8902@gmail.com; 2 Center of Biomedical Data Science (CBDS), Yonsei University Wonju College of Medicine, Wonju 26426, Korea; sam0246@naver.com (H.S.K.); dr.kang@yonsei.ac.kr (D.R.K.); 3 Department of psychiatry, Yonsei University Wonju College of Medicine, Wonju 26426, Korea

**Keywords:** non-daily smoking, daily smoking, tobacco control, mental health

## Abstract

Introduction: Non-Daily Smoking (NDS), which is increasingly prevalent worldwide, has not yet attracted as much attention as has daily smoking in Asia. The aims of this study were to identify trends in the prevalence of NDS and to compare characteristics by age, gender, and mental health indicators such as depression, suicidality, and alcohol consumption in South Korea. Methods: We included 33,806 adults (aged ≥ 19 years) who participated in the Korean National Health and Nutrition Examination Survey (KNHNES) from 2010 to 2015. The dataset includes self-reported medical history and questionnaires that explore depression, suicidality, and alcohol use, which are known to be highly related to smoking. We divided the respondents into four groups according to smoking status: Never Smoking (NS, N = 20,270); Past Smoking (PS = 6835); Daily Smoking (DS = 5927), who reported smoking every day; and Non-Daily Smoking (NDS = 774), who reported that they sometimes smoke. Results: Increased NDS prevalence is observed in most age groups in both male and female adults despite the prevalence of total smoking and daily smoking gradually decreasing. Depression and suicidality were significantly more prevalent in the NDS than the NS group (Depression Odds ratio, OR = 1.72, 95% Confidence interval, CI = 1.31–2.26; Suicidality OR = 3.14, 95% CI = 1.40–7.02). NDS is also associated with a higher frequency of binge drinking and alcohol use disorder than NS (OR = 4.17, 95% CI = 3.49–4.99). Conclusions: This study suggests that more concern is warranted for NDS given the increasing prevalence and characteristics of poor mental health in NDS respondents.

## 1. Introduction

Tobacco control is being strengthened globally, and has had the effect of reducing the smoking rate. At the same time, however, Non-daily smoking (NDS; or intermittent, occasional smoking) is emerging as a new problem [1,2]. NDS accounts for 16% to 33% of all smokers in the United States [3,4], with a high rate in the youth, college students, women, and ethnic minorities [3,5,6]. In Asia, 2% to 3% of all adults and about 5% of men engage in NDS [7,8,9,10]. Still, the majority of studies on smoking in Asia have not paid much attention to this group.

Recently, tobacco control has been strengthened in Asian countries. In the past decade, Korea has implemented various tobacco control policies, such as the expansion of anti-smoking support programs, strengthening of regulations on tobacco advertising, expansion of smoking cessation areas, and sharply raising cigarette prices, according to the World Health Organization Framework Convention on Tobacco Control recommendation [11]. Thanks to these policies, Korea’s overall adult smoking rate has decreased significantly, from 35.1% in 1998 to 22.4% in 2018 [12]. However, other countries that previously implemented such policies also have difficulty in reducing the prevalence of NDS [2]. Thus, there is a growing need to investigate the prevalence and characteristics of NDS and to design appropriate interventions.

One important reason to pay attention to NDS is that the majority of those who engage in NDS are not interested in specific smoking cessation treatments and seem to believe they could simply quit smoking at will [13]. Some individuals have no desire to quit smoking because they incorrectly identify themselves as non-smokers [13]. In fact, they often fail to quit even though they show lower levels of nicotine dependence than do those who engage in daily smoking (DS) [14]. A large number of them are social smokers who do not smoke on their own or with family, but only at parties or with friends [6,13,15], and often report that they smoke only while drinking [13]. Because of these characteristics, they do not think of themselves as smokers and tend to underestimate the effects that smoking has on them even though they are aware of the dangers of smoking in general [13,16,17].

There is some evidence that NDS is related to poor mental health. Historically, the relationship between smoking and depression and suicide has been studied primarily in DS. However, a growing body of evidence has shown that NDS and depression associated with each other [17,18] as well as suicidal ideation and suicide attempts, similar to DS. There are even reports of increasing depression among occasional smokers [19]. Furthermore, many studies point out that drinking and smoking are closely related, and this association is also observed with NDS [17,20,21,22,23,24]. Those who engage in NDS are more likely to smoke when drinking alcohol, and the desire for nicotine or the pleasure gained from smoking is greater than that of DS when drinking alcohol [20,23]. In addition, NDS is associated with a heightened risk of excessive alcohol use, similar to DS [17] or even higher [23]. However, the majority of studies are focused on college students and young adults. It is necessary to confirm the mental health risks in the whole adult population.

Therefore, we aimed to investigate the prevalence of NDS and to investigate its characteristics using data from the Korea National Health and Nutrition Survey. In particular, the annual trends for the six years from 2010 to 2015 were examined to understand how NDS was influenced by government smoking policy. In addition, the prevalence of decline in major mental health variables in the NDS group among all adults was identified in order to promote social interest in the risk of NDS.

## 2. Methods

### 2.1. Participants

The present study used a total of 6 years’ worth of raw data from the 5th (2010–2012) and the 6th (2013–2015) Korea National Health and Nutrition Examination Surveys (KNHANES). KNHANES is implemented to understand the health and nutritional status of the Korean people based on the National Health Promotion Act; it provides statistical data on smoking, drinking, physical activity, and obesity as requested by the World Health Organization (WHO) and the Organization for Economic Cooperation and Development (OECD).

Data from 5th and 6th KNHANES surveys were collected from 48,482 survey participants through a household visit interview. A total of 33,806 adults aged 19 years or older who completed the descriptions of smoking habits were included in the present study. We divided them into four groups according to smoking status: Never Smoking (NS, N = 20,270), who said they had not ever smoked in their lifetime; Past Smoking (PS = 6835), who smoked in the past but did not smoke at present; Daily Smoking (DS = 5927), who responded that they smoke every day; and Non-Daily Smoking (NDS = 774), who answered that they sometimes smoke.

### 2.2. Variables

#### 2.2.1. Socio-Demographics

The participants’ gender, age, education level, and subjective health status were collected. Education level was classified as “≤6 (below elementary school)”, “>6–≤9 (junior high school)”, “>9–≤12 (high school)”, and “>12 (higher education)”. Subjective health status variables were used to assess the respondents’ subjective health status, and they were asked to choose among five options for each variable: very good, good, moderate, poor, and very poor.

The questionnaire included an assessment of how often the participants drank alcohol in the last year: less than once a month; 1 to 4 times a month; 2 to 3 times a week; or 4 times a week or more. Binge drinking was measured by asking how frequently participants consumed at least 7 standard alcohol units (or 5 cans of beer) in a single session, and the results were divided into “never”, “less than once a week”, and “more than once a week”.

Smoking cessation plans were assessed only for those in the DS and NDS groups. Participants were asked whether they planned to quit in the future, and they were given a choice of four options: “I plan to quit smoking within one month”, “I plan to quit smoking within 6 months”, “I am going to quit smoking sometime but not within 6 months”, and “I don’t have any plan to quit smoking”.

#### 2.2.2. Mental-Health-Related Variables

In the present study, we examined data on depression, suicidality, and alcohol use, which are known to be highly related to smoking. For depression, history of depression and current depression were evaluated. For history of depression, the participants were asked whether they had experienced depressive feelings that disrupted daily life for more than two weeks continuously over the previous year. For current depression, the participants were asked whether they are currently suffering from depression as diagnosed by a physician.

Suicidal thoughts, suicide planning, and suicide attempts in the previous year were identified. The participants answered yes or no to the following questions: “Have you ever thought about suicide seriously in the last year?” “Have you made a specific plan to commit suicide in the last year?” and “Have you actually tried suicide in the last year?”

Pathological drinking was evaluated using the Alcohol Use Disorder Identification Test (AUDIT). AUDIT is a self-reported questionnaire composed of 10 questions developed by the WHO. Each question is graded on a 4-point Likert scale for a total score ranging from 0 to 40 points. It is most effective at screening for dangerous drinking, problematic drinking, and alcohol use disorder. In this study, cut-off values of 11 points for males and 7 points for females were applied [25].

### 2.3. Statistical Analysis

The chi-square test was used to confirm differences between the four groups according to smoking status for demographic categorical variables: gender, education level, subjective health status, drinking frequency, and smoking cessation planning. One-way ANOVA was also performed to identify age differences between the groups.

To determine the prevalence of smoking in the general population, an incidence frequency (count) was calculated by applying the weights of each year to smoking status. A crude rate (CR) was calculated using information from the population census or the person year (PY) from the National Statistical Office. The formulas used to calculate CR and 95% Confidence interval (CI) are as follows.

(1) $$\mathrm{CR}=\frac{Count\;}{PY}$$

(2) $$95\%\;\mathrm{CI}=\mathrm{CR}\pm 1.96\times \sqrt{\frac{Count}{PY}}$$

The CR was calculated by age group in 10-year brackets. The age-adjusted rate (AR) was obtained by calculating the weight (W) for each age range according to the age structure of the 2010 census data. The calculation formula is as follows.

(3) $$\mathrm{AR}=\underset{age\;range_{i}}{\sum }\left(W_{i}\times CR_{i}\right)$$

(4) $$\mathrm{with}\;W_{i}=\frac{PY_{i}}{\sum^{}PY_{i}}$$

(5) $$95\%\;\mathrm{CI}=\mathrm{AR}\pm 1.96\times \sqrt{Var\left(AR\right)}$$

(6) $$\mathrm{with}\;\mathrm{Var}\left(\mathrm{AR}\right)=\sum^{}W_{i}^{2}\times \frac{Count_{i}}{{\left(PY_{i}\right)}^{2}}$$

Trends in smoking prevalence from 2010 to 2015 were identified by sex and age. In addition, a two-way ANOVA was performed to compare the prevalence between groups, between times, and interaction effect of the two factors. Since the mental health-related variables “depression experience,” “current depression,” “suicidal thoughts,” “suicide plan,” “suicide attempt,” and “AUDIT” are binary, logistic regression was performed to examine the influence of smoking on the individual variables. We report an odds ratio (OR) of model adjusted for sex and age, which are generally known to affect smoking status, and also revealed differences between groups. The significance level was set at 0.05. The mental health-related variables were investigated for the years 2010–2013 and 2015, excluding 2014. Therefore, we analyzed data from a total of 28,614 subjects (DS = 5042, NDS = 662, PS = 5838, NS = 17,072) for the mental health-related variables in the years 2010–2013 and 2015. SAS 9.4 (SAS Inc, Cary, NC, USA) and SPSS 23.0 (IBM, Armonk, NY, USA) were used for data analysis.

## 3. Results

### 3.1. Socio-Demographics

Table 1 shows classification according to smoking status. Of 33,806 adults, 5927 were in the DS group, 774 were in the NDS group, 6835 were in the PS group, and 20,207 were in the NS group. There was a significant difference in sex, age, and education level between all groups (*p* < 0.001). NDS had the lowest average age and highest educational level of all groups. On the other hand, the NDS group had significantly worse subjective health status than did the NS group (*p* < 0.002). Regarding alcohol use, the NDS group reported a lower drinking frequency and binge drinking frequency than the DS group (drinking frequency *p* < 0.002; binge drinking frequency *p* < 0.001). However, NDS was associated with significantly higher drinking frequency than NS (*p* < 0.002) and higher binge drinking frequency than PS and NS (NDS-PS *p* < 0.001, NDS-NS *p* < 0.001). There was also a significant difference between the NDS and DS groups in planning for smoking cessation (*p* < 0.001). Those in the NDS group most frequently reported having a plan to quit “within one month” (52.84%), while the DS group most often reported “someday but not within six months” (36.97%) or “no plan to quit” (30.59%).

### 3.2. Annual Trends in Smoking Prevalence

**Figure 1:**
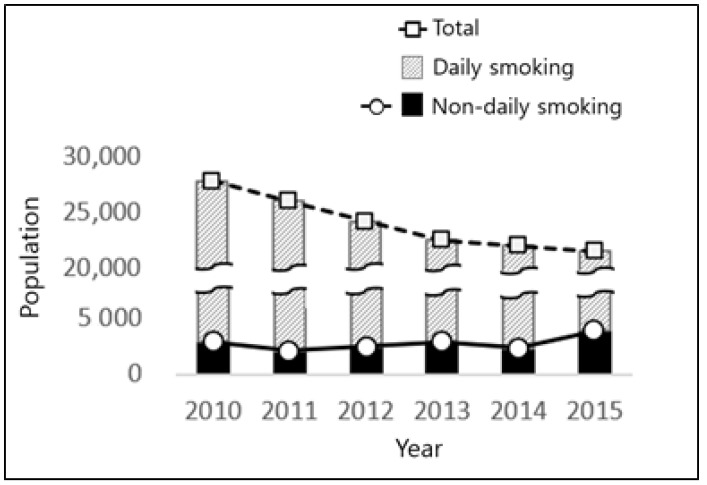
Annual trends of smoking population in the Korean.

**Figure 2:**
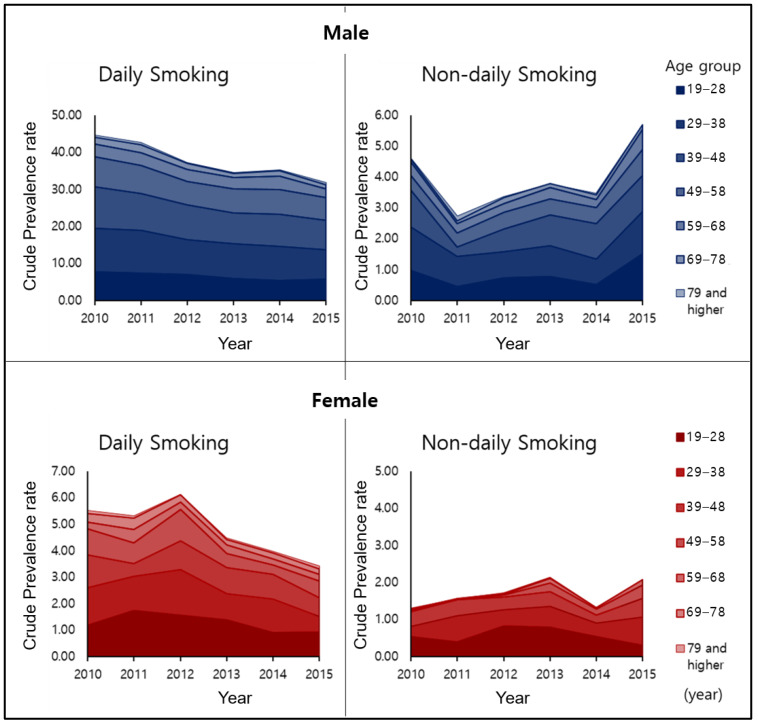
Crude prevalence of smoking by age and sex from 2010 to 2015.

The overall trends in the number of smokers in Korea from 2010 to 2015 are shown in Figure 1. The prevalence of total smoking and DS decreased markedly, but NDS increased steadily, except in 2010, and increased significantly in 2015. Figure 2 and Table 2 show the prevalence of smoking by age and sex. There was a temporary increase in female smoking in 2012 in the DS group, but both males and females showed an overall decline. However, in the case of NDS, males have shown a steady upward trend since 2011, resulting in an increase in prevalence in 2015 compared to 2010 (2010 = 4.61%, 2015 = 5.70%). Similarly, the overall trend in female NDS increased from 2010 to 2015 (2010 = 1.29%, 2015 = 2.06%), though there was a temporary decline in 2014. Two-way ANOVA for prevalence showed that the interaction effect between group and time was significant in both genders (Male: F = 36.61, *p* = 0.003; Female: F = 11.42, *p* = 0.010). Additionally, the main effect of group (F = 591.16, *p* < 0.001) and time (F = 46.99, *p* = 0.001) was found to be significant in male. In females, the main effect of the group was statistically significant (F = 11.49, *p* = 0.010). That is, the difference in prevalence between groups changed over time in both genders. The nature of change is indicated by a decrease in DS and an increase in NDS in males and decrease in DS in females.

The increase in NDS prevalence is observed in most age groups in both sexes. For male NDS, the increasing tendency is seen in all age groups except for people aged 69 and over (Table 2). This tendency is similar in females: in the middle years, between 29 and 69, there was a significant increase in 2015 compared to 2013, though young adults (19–28) had the highest prevalence in 2012, followed by a gradual decline thereafter.

### 3.3. Mental Health

Table 3 shows the results of logistic regression analysis on the effects of smoking status on mental health, adjusted for age and gender. First, in depression-related variables, NDS is associated with an increased risk compared to NS and PS. In the NDS group, the risk of having experienced depression was significantly elevated (OR = 1.72, 95% CI = 1.31–2.26) and the risk of current depression was also significantly higher than in the DS group. (NDS OR = 1.73, 95% CI = 1.00–3.00; DS OR = 1.43, 95% CI = 1.08–1.89). Second, for suicide-related variables, the risk of suicidal ideation was significantly higher in the NDS than in the NS group (OR = 1.32, 95% CI = 1.011.74). Additionally, the risk of suicide planning was also increased in the NDS group, and it was similar to that seen in the DS group (NDS OR = 3.14, 95% CI = 1.40–7.02; DS OR = 3.25, 95% CI = 1.99–5.29). The risk of attempting suicide was significantly increased only in the DS group (NDS OR = 2.02, 95% CI = 0.97–4.20; DS OR = 2.61, 95% CI = 1.80–3.80). Finally, the risk of alcohol use disorder was significantly higher in the NDS group than in the NS, PS, and DS groups (PS OR= 2.28, 95% CI = 2.09–2.49; NDS OR= 4.17, 95% CI = 3.49–4.99; DS OR = 3.93, 95% CI = 3.59–4.30).

## 4. Discussion

We identified the prevalence and the characteristics of NDS by analyzing data from the National Health and Nutrition Survey and identified trends in NDS prevalence from 2010 to 2015. In addition, we explored the relationship between NDS and mental-health-related variables.

In the present study, NDS was present in about 2.1–3.9% of the total population and accounted for about 8–38% of total smokers, which is similar to the rate seen in other countries [4,7,8,9,10]. The prevalence of NDS is steadily increasing, while that of DS and total smoking is decreasing in most parts of all age groups. This trend is similar to that seen in Europe and North America, which currently have a relatively high prevalence of NDS. In the United States, only 5.8% of total adult smokers were in the NDS group in 1991, but that increased to 21.6% in 2012, while overall smoking rates declined from 25.2% to 18.1% over the same period [26,27].

This study found that NDS prevalence in Korea decreased slightly in 2011 and 2014. This may be related to stringent tobacco control policies. A ban on tobacco advertisements and a mandate for smoke-free public places began in 2011, and tobacco tax increases were announced in 2014 [28]. Although the price of cigarettes remains low considering the nation’s high average income, an 80% price increase, which had not been done for 10 years prior, would have been a significant burden. The prevalence of DS in 2015 compared to 2010 indicates that current Korean tobacco control policies have been effective in reducing DS and the total smoking rate; however, the rate of NDS did not decline over the same time period, and instead increased. There is some indication that some individuals in the DS group convert to the NDS group following smoking cessation attempts [29], which could suggest that the decrease in DS and the increase in NDS seen in this study may be related to smoking cessation attempts. It is difficult to draw a firm conclusion as to whether an increase in NDS is associated with a decrease in smoking intensity or an increase in the use of nicotine alternatives since this is a cross-sectional study. Regardless, smoking cessation policies should take NDS into account and specific interventions should be explored.

NDS represents a significantly different pattern of cigarette craving than DS. Nicotine dependence is less common, so smoking behavior is more likely to be driven by secondary motives such as seeking out positive psychological stimuli or weight control [30]. These are similar to women’s motives for smoking [31,32,33]. Considering the differences in motivation, smoking cessation strategies for NDS may have to differ from those made to address DS, just as strategies to help female smokers are different from those tailored towards men [34]. Individuals who engage in NDS are also strongly influenced by social situations in their smoking behavior [15,24]. Therefore, smoking cessation strategies for NDS need to be distinguished from those for DS.

The present study shows that gender differences in the prevalence of NDS and total smoking are more prominent in Korea than in Europe or North America [35,36]. In Asia, smoking is considered to be a masculine trait, and female smoking tends to be seen as undesirable. Korean women especially avoid revealing themselves as smokers [36,37]. However, the self-reported smoking rate rises significantly when confidentiality is guaranteed [36,38], and the smoking rate more than doubles when assessed by urinary cotinine testing [39]. So, it is possible that the rates of NDS and DS among women in Korea are higher than the results of this study would indicate. However, it is noteworthy that, as the price of cigarettes increases, the female NDS temporarily decreases and then increases in the following year, like the male NDS change.

As in previous studies, we found that NDS is associated with a higher risk of depression and suicidality than NS. Smoking and depression frequently co-occur and have several common underlying psychological factors [40,41,42]. Both DS and NDS are closely related to depression [19,43,44], and NDS as well as DS are significantly more likely to be associated with suicidal ideation than NS [45]. In terms of its relationship to mental health, such as depression and suicidality, there is a need to improve public awareness of the risks of NDS.

The results of the present study also suggest that NDS is associated with a high risk of alcohol use disorder as well as a high frequency of binge drinking. These results are consistent with previous studies [17,46,47]. Drinking is a risk factor for cigarette smoking and a major impediment to cessation. In particular [48,49], NDS leads to more intense feelings of nicotine-related craving and pleasure than DS when alcohol is consumed [20,23]. Therefore, it is necessary to actively intervene more in cases of NDS accompanying substance use disorder.

The limitations of this study are as follows. First, although the trend of change was examined with data from a six-year NDS survey, it was not possible to examine changes made by individuals who partook in NDS because the data did not allow for continuous follow-up of single subjects. Therefore, future studies need to explore individual trajectories and develop a smoking cessation strategy appropriate for each lifestyle. Second, this study was useful for exploring the general characteristics of NDS populations using a large data set, but it was difficult to identify specific behavioral or psychological characteristics. In particular, since cross-sectional data was used, it was difficult to draw causal conclusions about smoking and mental health variables such as depression. Third, smoking and characteristics such as depression, suicide, and alcohol use were self-reported and not biochemically or objectively confirmed. Additionally, the dataset used in this study took a brief medical history of depression and suicidality without using a standardized questionnaire. Fourth, this study is a secondary data analysis of nationally representative health surveys, but data weighting was not applied to estimate the prevalence [50]. Standard statistical analyses that do not use weighted multiple complex survey data such as generally (1) yield biased point estimates of population parameters, (2) underestimate the standard error for point estimates, (3) produce overly-narrow confidence intervals on population parameters, and (4) yield tests of significance that are overly likely to reject the null hypothesis because standard error or variability of statistics is generally underestimated, resulting in more frequent type I errors [51]. Therefore, we need to incorporate sample weights, stratification, and clustering of the design into the analysis to ensure the development of appropriate estimates and standard errors of these estimates. Finally, this study does not reflect the use of nicotine substitutes, including e-cigarettes, which may be associated with increased NDS. We attempted to investigate the total smoking rate as much as possible, but the survey items on the use of e-cigarettes were only included beginning in 2013, so they were excluded from this study. However, it was found that only 0.4% of nicotine users reported using only electronic cigarettes [52].

## 5. Conclusions

This study used large-scale data to identify the prevalence and changing trends of NDS which have been relatively unexamined in Asia to date. In addition, the relationship between NDS and mental health variables was explored to confirm the risk of NDS. Tobacco control policies in Korea have been shown to be effective in reducing DS prevalence, but not very effective in reducing NDS. Moreover, NDS has been shown to be closely related to mental health problems such as depression and alcohol use. This study would be useful for developing awareness of the continued increase in and risks associated with NDS and raising interest in the development of appropriate smoking cessation strategies.

## Figures and Tables

**Table 1:** Socio-demographic variables.

Characteristics	DS ^a^ (n = 5927)	NDS ^b^ (n = 774)	PS ^c^ (n = 6835)	NS ^d^ (n = 30,371)	*p*-Value
Sex (Male)	5145 (86.81)	520 (67.18)	5779 (84.55)	2887 (14.24)	<0.001 ^†^
Age	46.77 ± 15.22	43.22 ± 15.28	55.23 ± 16.14	50.47 ± 16.83	<0.001 (b < a < d < c)
Education					<0.001 ^†^
≤6 years	960 (16.61)	70 (9.32)	1522 (22.78)	5617 (28.24)	
>6–≤ 9 years	638 (11.04)	80 (10.65)	834 (12.49)	2016 (10.14)	
>9–≤ 12 years	2340 (40.50)	302 (40.21)	2155 (32.26)	6336 (31.86)	
>12 years	1840 (31.84)	299 (39.81)	2169 (32.47)	5918 (29.76)	
Perceived health status					<0.001
Very good	231 (3.90)	37 (4.78)	374 (5.47)	3135 (10.32)	b–a (0.028), b–c (0.023), b–d (0.002)
Good	1434 (24.19)	218 (28.17)	1976 (28.91)	10,215 (33.63)	
Average	3043 (51.34)	373 (48.19)	3126 (45.74)	12,243 (40.31)	
Poor	904 (15.25)	111 (14.34)	952 (13.93)	3567 (11.74)	
Very poor	179 (3.02)	13 (1.68)	266 (3.89)	857 (2.82)	
Drinking					<0.001
<1/month	1335 (22.52)	149 (19.25)	2264 (33.12)	12,262 (60.49)	b–a (0.002), b–c (0.023), b–d (0.002)
1–4 times/month	1938 (32.70)	357 (46.12)	2314 (33.86)	6058 (29.89)	
2–3 times/week	1677 (28.29)	194 (25.06)	1385 (20.26)	1491 (7.36)	
≥4 times/week	969 (16.35)	70 (9.04)	851 (12.45)	410 (2.02)	
Binge drinking					<0.001 ^†^
Never	1826 (30.81)	272 (35.14)	3234 (47.32)	16,657 (82.18)	
<1/month	1740 (29.36)	246 (31.78)	1921 (28.11)	2686 (13.25)	
>1/week	2358 (39.78)	255 (32.95)	1671 (24.45)	922 (4.55)	
Smoking cessation plan					<0.001
Within a month	1063 (17.93)	409 (52.84)	-	-	
Within 6 months	845 (14.26)	107 (13.82)	-	-	
Someday but not within 6 months	2191 (36.97)	160 (20.67)	-	-	
No plans to quit	1813 (30.59)	96 (12.40)	-	-	

DS: Daily Smoking, NDS: Non-Daily Smoking, PS: Past Smoking, NS: Never Smoking. Signs ^a^, ^b^, ^c^, and ^d^ mean DS, NDS, PS and NDS, respectively. Data are given as mean ± standard deviation or numbers and percentages; ^†^: Statistical significance in group comparisons of all possible combinations.

**Table 2:** Annual age-adjusted smoking prevalence.

Age Group (Year)	Male						Female					
	2010	2011	2012	2013	2014	2015	2010	2011	2012	2013	2014	2015
19~28												
DS	7.68	7.34	6.91	5.96	5.36	5.74	1.19	1.73	1.56	1.38	0.90	0.92
NDS	0.96	0.44	0.73	0.77	0.51	1.50	0.53	0.39	0.83	0.80	0.54	0.29
29~38												
DS	11.73	11.54	9.50	9.28	9.23	7.89	1.40	1.30	1.72	0.98	1.28	0.60
NDS	1.43	0.98	0.84	1.00	0.82	1.37	0.28	0.71	0.44	0.55	0.36	0.77
39~48												
DS	11.26	10.01	9.40	8.44	8.75	7.91	1.24	0.48	1.09	0.98	0.93	0.69
NDS	1.18	0.30	0.73	1.01	1.15	1.16	0.38	0.45	0.34	0.40	0.22	0.51
49~58												
DS	8.12	7.49	6.38	6.39	6.52	6.14	0.99	0.79	1.20	0.54	0.35	0.64
NDS	0.48	0.45	0.55	0.51	0.52	0.84	0.05	0.00	0.06	0.23	0.15	0.35
59~68												
DS	3.51	3.43	3.12	3.09	3.75	2.47	0.25	0.51	0.28	0.33	0.22	0.26
NDS	0.44	0.31	0.29	0.38	0.27	0.65	0.02	0.00	0.02	0.12	0.03	0.14
69~78												
DS	1.79	2.22	1.74	1.14	1.40	1.11	0.32	0.41	0.26	0.19	0.22	0.20
NDS	0.09	0.10	0.18	0.12	0.16	0.16	0.02	0.00	0.03	0.01	0.00	0.00
Total	49.24	45.47	40.66	38.38	38.76	37.5	6.82	6.89	7.84	6.62	5.32	5.49
DS	44.63	42.73	37.29	34.59	35.28	31.80	5.53	5.32	6.13	4.49	3.99	3.43
95% CI	44.60–44.66	42.70–42.76	37.26–37.32	34.56–34.62	35.25–35.31	31.78–31.83	5.51–5.54	5.31–5.33	6.12–6.14	4.48–4.50	3.98–4.00	3.42–3.44
NDS	4.61 (9.36)	2.74 (6.03)	3.37 (8.29)	3.79 (9.87)	3.48 (8.98)	5.70 (15.20)	1.29 (18.91)	1.57 (22.79)	1.71 (21.81)	2.13 (32.18)	1.33 (25.00)	2.06 (37.55)
95% CI	4.60–4.62	2.73–2.75	3.36–3.38	3.78–3.79	3.47–3.49	5.69–5.71	1.29–1.30	1.57–1.58	1.70–1.71	2.12–2.13	1.32–1.33	2.06–2.07

DS: Daily Smoking, NDS: Non-Daily Smoking, CI: Confidence interval.

**Table 3:** The effect of smoking status on mental health according to a logistic regression model.

OR (95% CI)						
	Depression Experience ^†^	Current Depression	Suicide Ideation ^†^	Suicide Planning ^†^	Suicide Attempt ^†^	Alcohol Use Disorder
NS	1.00	1.00	1.00	1.00	1.00	1.00
PS	1.46 ** (1.27–1.67)	1.14 * (0.86–1.50)	1.48 ** (1.30–1.69)	2.17 ** (1.34–3.53)	1.45* (0.95–2.19)	2.28 ** (2.09–2.49)
NDS	1.72 ** (1.31–2.26)	1.73 * (1.00–3.00)	1.32 * (1.01–1.74)	3.14 ** (1.40–7.02)	2.02 * (0.97–4.20)	4.17 ** (3.49–4.99)
DS	2.08 ** (1.81–2.39)	1.43 ** (1.08–1.89)	2.38 ** (2.09–2.71)	3.25 *** (1.99–5.29)	2.61 ** (1.80–3.80)	3.93 ** (3.59–4.30)

Model adjusted for age and sex. DS: Daily smoking, NDS: Non-daily smoking, PS: past smoking, NS: never smoking; OR: odds ratio, CI: confidence interval; * *p* < 0.05, ** *p* < 0.01, *** *p* < 0.001; ^†^: 2014 data not available.
